# Primary Tuberculous Pyomyositis of Quadriceps Femoris in an Immunocompetent Individual

**DOI:** 10.1155/2013/723879

**Published:** 2013-12-03

**Authors:** M. A. Modi, A. D. Mate, A. M. Nasta, A. K. Gvalani

**Affiliations:** Department of General Surgery, Seth GS Medical College and KEM Hospital, Parel, Mumbai 400012, India

## Abstract

Primary tuberculous pyomyositis is a rare manifestation of musculoskeletal tuberculosis especially in immunocompetent individuals without a focus of tuberculosis in the body and the underlying bone disease. It can cause a diagnostic dilemma for a physician and surgeon because of its similar presentation to soft tissue sarcomas, hematomas, and myopathies. We present a case of a 45-year-old immunocompetant gentleman with a thigh swelling with sepsis due to pyomyositis of the quadriceps requiring a multimodal management of drainage of abscess, debridement of devitalized muscle, antitubercular drugs, and physiotherapy. In a tubercular endemic country, a high index of suspicion is required to diagnose this disease which can be cured completely.

## 1. Introduction

Musculoskeletal tuberculosis occurs in only 3% of patients with tuberculosis, mostly presenting with spondylitis, osteomyelitis, or arthritis [1]. Primary tuberculous pyomyositis is a rare entity constituting less than 1% of skeletal tuberculosis [2]. This can mimic inflammatory myositis or rarely cancer and can create diagnostic confusion for surgeon. It should always be considered as a rare but possible aetiology of myositis [3]. Tubercular myositis in an immunocompetent patient without underlying bony involvement is an unusual presentation and its pathogenesis is still unclear [4, 5]. We present a case of primary tubercular pyomyositis of the quadriceps femoris.

## 2. Case Presentation

**Figure 1:**
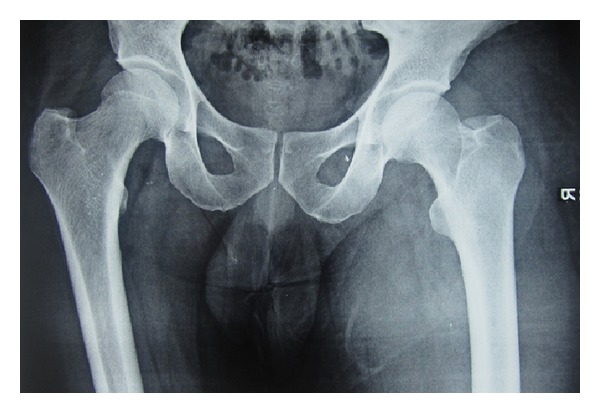
X-ray bilateral hip joint—X-ray showing a normal hip joint and femur with soft tissue swelling on right thigh.

**Figure 2:**
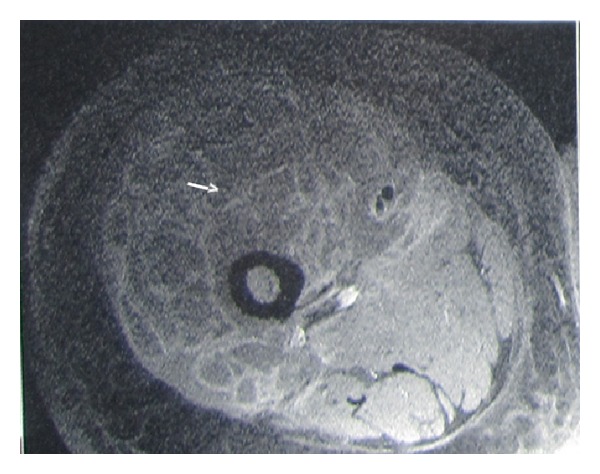
MRI right thigh—MRI axial T1W image—showing a multiseptated intramuscular abscess with debris in quadriceps femoris.

**Figure 3:**
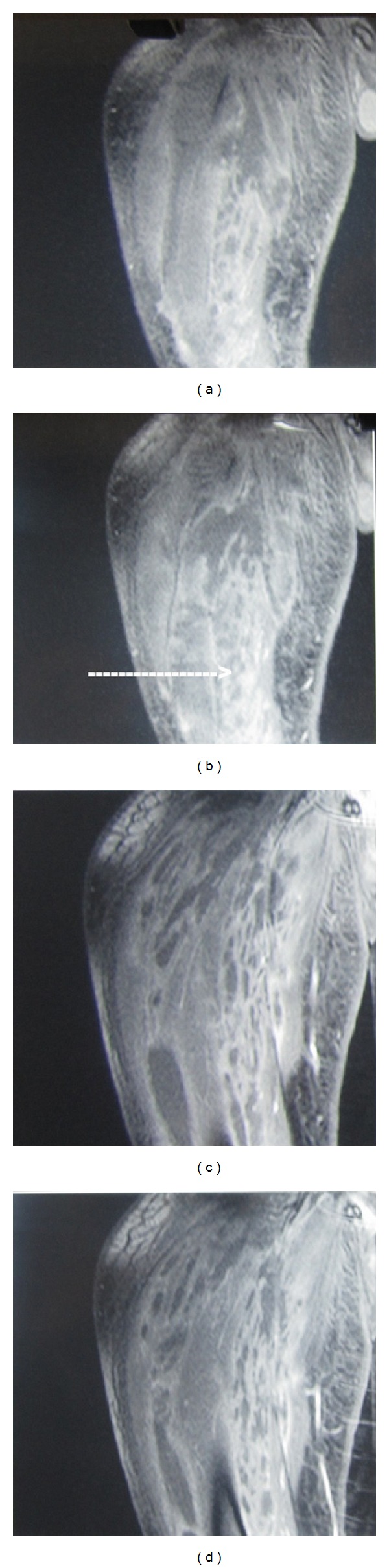
MRI right thigh—MRI coronal T1W—showing a diffuse septated intramuscular collection suggestive of pyomyositis of quadriceps femoris.

**Figure 4:**
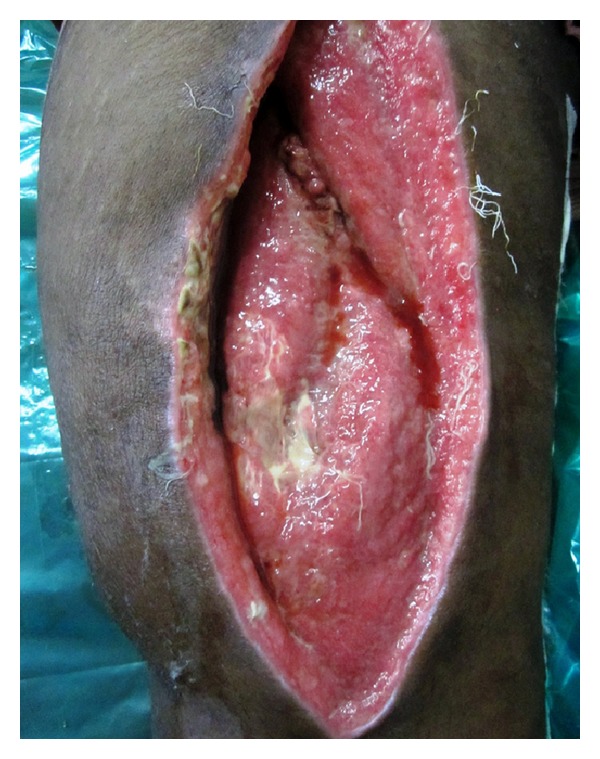
Wound after debridement—granulating wound on right thigh (anterior view).

**Figure 5:**
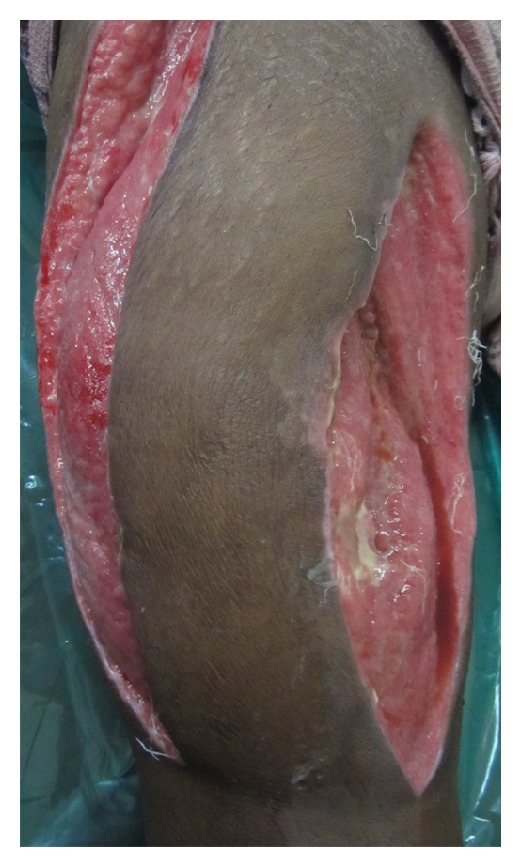
Wound after debridement—granulating wound on right thigh (lateral view).

A 45-year-old male patient presented with an 8-day history of painful, progressively increasing swelling of the right thigh. There was no history of fever, weight loss, and anorexia. There was no preceding history of any trauma, diabetes, immunosuppression, corticosteroid usage, or renal failure and no past history of tuberculosis or contact. On examination, patient had tachycardia. The right thigh was diffusely swollen, tender, firm, and warm without any scars or sinuses. Distal neurovascular status was normal. There was no lymphadenopathy, spinal tenderness, or paraspinal swelling. The movements of the hip and knee joint were normal (Figure 1). Radiographs of the chest, ipsilateral femur with hip, and knee joint were normal. Doppler ultrasound showed normal deep venous system with subcutaneous edema over thigh associated with myositis. There was no evidence of pus collection in subcutaneous as well as subfascial compartments. Blood investigations including CBC and blood sugar were normal. ESR was elevated at 96 mm/hr. HIV-ELISA was negative. Creatinine kinase was slightly elevated at 500 IU/L. The patient was managed conservatively with intravenous antibiotics covering gram-positive and anaerobic organisms with inflammatory myositis as the working diagnosis. In view of persistent tachycardia, rising counts and fever pyomyositis were suspected. An MRI of right thigh was performed and pyomyositis of the quadriceps femoris was confirmed (Figures 2 and 3). The intramuscular abscess was drained, necrotic muscle debrided. Smear from pus showed the presence of pus cell and acid fast bacilli. TBM-PCR-DNA of the pus was positive. Culture could not isolate any organism. Histopathology from involved muscles revealed inflammatory cells and granulomatous cells suggestive of tuberculosis. Patient was started on four-drug antitubercular treatment (2HREZ/4HR) and regular dressings. The patient's general condition improved and the wound granulated eventually being covered with a graft (Figures 4 and 5). The patient completed 6 months of antitubercular treatment. On a two-year followup, the patient is asymptomatic with a scar and a flexion contracture of the knee as the residue of the disease.

## 3. Discussion

Extra pulmonary infection accounts for 40% of all tuberculosis cases [3]. Common sites of extra pulmonary tuberculosis are lymph nodes and abdomen. Musculoskeletal tuberculosis occurs only in 3% of cases. Common forms of musculoskeletal tuberculosis are spondylitis, osteomyelitis, or arthritis [2]. Tuberculous myositis is extremely rare, with an estimated incidence of 0.015−2% of extra pulmonary involvement [6].

The few case reports in the literature about tubercular pyomyositis are in immunodeficient, HIV infected, and renal failure patients or in patients on corticosteroids, immunosuppressive drugs, or chemotherapy [7–10]. It also has been described in immunocompetent patients within different muscles [11–14].

Commonest route of involvement is by contagious spread (63% cases). Haematogenous spread accounts for 29% of cases. In 8% of the cases cause is direct inoculation. The most common sites of involvement are paraspinal and chest wall muscles by contagious spread [2]. Involvement of thigh muscles is rare and is usually limited to one muscle, principally quadriceps femoris, and spread is usually haematogenous in case of a primary focus elsewhere [6, 15].

Tubercular pyomyositis in the absence of direct spread from underlying bony lesion or haematogenous spread could occur in an immunocompromised patient [16], by direct inoculation [17] or idiopathic as in our case.

Skeletal muscles are considered to be “forbidden tissue” for growth and multiplication of tubercle bacilli [5] because of poor oxygen content, high lactic acid concentration, and paucity of reticuloendothelial cells [18]. Tubercular bacilli do not produce proteolytic enzymes; hence, it does not cause a pyogenic infection, but it may get secondarily infected leading to abscess formation in surrounding tissue [19] which seems to be the case in our patient.

Clinical manifestations are usually nonspecific. This might lead to diagnostic dilemma. Tubercular pyomyositis is commonly misdiagnosed as a soft tissue sarcoma, parasitic infection like cysticercosis or hydatid cyst, and inflammatory myositis or hematoma with secondary infection because of its close resemblance clinically [5]. There is usually a delay in diagnosis because of its atypical presentation, lack of knowledge of the disease, absence of early specific signs, and a large number of differentials. High index of clinical suspicion is the key in diagnosis especially in endemic areas [2].

Blood investigations usually are normal except a raised ESR which is a consistent finding [5].

DNA-PCR is a highly sensitive investigation especially to differentiate tubercular from nontuberculous mycobacteria which cause soft tissue infection. With its multiplanar capability and contrast for soft tissue, MRI is the investigation of choice. Most cases of M. tuberculosis myositis are initially diagnosed as bacterial pyomyositis. Although it is difficult to differentiate between M. tuberculosis and Staphylococcal pyomyositis in the early stage of disease, MRI findings can help to distinguish between them. On MRI exam, M. tuberculosis myositis is characterized by low signal intensity on T1WI and high signal intensity on T2WI in a single muscle, with peripheral rims showing subtle hyperintensity on T1WI and hypointensity on T2WI. After gadolinium infusion, peripheral rim enhancement is observed in all cases [3]. Unlike pyogenic myositis, neither cellulitis nor venous thrombosis surrounded the involved muscle [2]. MRI may be useful in the management of tubercular myositis in delineating the anatomical extent of muscle lesions and guiding the surgeons in debridement. MRI is superior to CT or USG in the detection and characterization of the swelling in order to differentiate it from malignancy [2, 20].

This report is unique because primary tubercular pyomyositis of the quadriceps has been reported only on three occasions—Abdelwahab et al. [2], Simon et al. [9], and Bhatty et al. [21]. In Simons' study the patient was immunocompromised. Bhatty et al. noted this occurrence in an immunocompetent patient who presented with a mass in the vastus lateralis but did not show signs of sepsis and was operated with the probable diagnosis of sarcoma. We present this case of a primary tubercular pyomyositis in an immunocompetent patient without any identifiable focus elsewhere in the body in a tubercular endemic country like India. MRI in contrast is the investigation of choice. Surgery should be performed in patients with pyomyositis with features of sepsis, and a high index of suspicion for tuberculosis must be present. The outcome was excellent with a multimodal approach of the drainage of abscess, debridement of devitalized tissue, and antitubercular treatment.
